# Object responses are highly malleable, rather than invariant, with changes in object appearance

**DOI:** 10.1038/s41598-020-61447-8

**Published:** 2020-03-13

**Authors:** Desiree E. Holler, Sara Fabbri, Jacqueline C. Snow

**Affiliations:** 1 Department of Psychology, University of Nevada, Reno, USA; 2 Department of Experimental Psychology, University of Groningen, Groningen, the Netherlands

**Keywords:** Psychology, Human behaviour, Cognitive neuroscience, Perception, Object vision

## Abstract

Theoretical frameworks of human vision argue that object responses remain stable, or ‘invariant’, despite changes in viewing conditions that can alter object appearance but not identity. Here, in a major departure from previous approaches that have relied on two-dimensional (2-D) images to study object processing, we demonstrate that changes in an object’s appearance, but not its identity, can lead to striking shifts in behavioral responses to objects. We used inverse multidimensional scaling (MDS) to measure the extent to which arrangements of objects in a sorting task were similar or different when the stimuli were displayed as scaled 2-D images, three-dimensional (3-D) augmented reality (AR) projections, or real-world solids. We were especially interested in whether sorting behavior in each display format was based on conceptual (e.g., typical location) versus physical object characteristics. We found that 2-D images of objects were arranged according to conceptual (typical location), but not physical, properties. AR projections, conversely, were arranged primarily according to physical properties such as real-world size, elongation and weight, but not conceptual properties. Real-world solid objects, unlike both 2-D and 3-D images, were arranged using multidimensional criteria that incorporated both conceptual and physical object characteristics. Our results suggest that object responses can be strikingly malleable, rather than invariant, with changes in the visual characteristics of the stimulus. The findings raise important questions about limits of invariance in object processing, and underscore the importance of studying responses to richer stimuli that more closely resemble those we encounter in real-world environments.

## Introduction

Understanding how naturalistic stimuli are processed and represented in the human brain remains a major challenge for psychology, neuroscience and computer vision. Current theoretical frameworks of object vision posit that effective recognition requires *invariance* –that mental representations remain stable despite changes in visual cues that can alter an object’s appearance but not its identity^1–5^. Support for representational invariance has been derived from behavioral^6–8^ and neuroimaging studies^3,9^ (although with some limitations, for example, depending on the retinal location of the stimulus^10,11^ and task demands^12^). Two-dimensional (2-D) images of objects have been shown to elicit size-invariant responses during recognition^13^, priming^7,14^, perceptual learning^15^, and visual search^16^. Similarly, neuroimaging studies have shown that responses in shape-selective regions within ventral occipito-temporal (vOT) cortex remain relatively constant despite changes in image size^17,18^, as well as changes in position^19^, viewpoint^4,9,11,15^ and depth cues^18,20^. Reliable adaptation in ventral object areas across changes in the size of object images is observed in children by 5 to 10 years of age^21^. FMRI responses in vOT have also been found to remain constant across changes in the format in which a stimulus is displayed, such as when objects are illustrated as line-drawings, shaded images or photographs^18,22,23^.

The possibility remains, however, that object responses may be influenced by changes in visual appearance that are difficult to convey using pictorial cues. Two-dimensional (2-D) images convey impoverished information about the egocentric distance and real-world size of the object because, from the perspective of the observer, only the distance to the projection surface is known, but not the distance to the object. The real-world size of the pictorial stimuli used in most studies of object perception is further obscured by the fact that items are presented without background context, and the retinal extent of objects that are typically large in the world (e.g., a hot air balloon) is scaled to match those that are smaller (e.g., a coin)^24–28^. In contrast, real-world solids convey unambiguous information about egocentric distance, physical size and weight. Although distance, size and weight cues rarely change an object’s identity, these characteristics can influence identification^13^ and goal-directed actions. Although it may seem intuitive that solid objects could trigger different processes to those of scaled two-dimensional (2-D) pictures of objects, this raises the question of why most studies of human vision use scaled planar images as stimuli, and whether they are equivalent proxies for their real-world counterparts.

Three-dimensional (3-D) stereoscopic images more closely approximate the visual appearance of real-world solid objects because depth cues from disparity convey information about apparent distance and size, as well as 3-D geometric shape. However, stereoscopic images differ from real objects in that they cannot be grasped with the hands. Recently, technological advances have paved the way for augmented reality (AR) systems that can project high-fidelity images onto a transparent head-mounted display (HMD) so that the 3-D stimulus appears to be situated within the observer’s physical environment. AR stimuli (like regular stereoscopic images) differ visually from real objects in that they do not provide reliable depth cues from vergence and accommodation. Nevertheless, some AR systems, which allow the user to interact with 3-D projections with the hands, can be used to present objects that look very similar to real-world objects with respect to visual appearance and they offer the potential for manual interaction, even though they are not physical solids. However, AR objects differ conceptually from real objects (as well as 2-D images) because in real-world environments and natural scene images the objects typically appear in specific locations^2,29^, whereas a defining characteristic of AR projections is that they can appear anywhere. For example, a real-world office can be ‘augmented’ with a virtual toothbrush, even though a toothbrush is not typically located in an office setting.

Here, we leveraged the similarities and differences between 2-D images, virtual AR projections and real-world solids, to test whether behavioral responses to objects remain stable when the stimuli convey different conceptual and physical characteristics. Although ferrous components in AR HMDs currently prevent their application in fMRI contexts, behavioral results can offer a window into the nature of underlying mental object representations, for example using inverse multidimensional scaling (MDS)^26,30^. MDS assumes a geometrical model of mental representation^31^ in which perceived similarities between objects reflect the relationship between those objects in a conceptual space that corresponds to the underlying mental representation^26,32^. Using this approach, observers are presented with an array of objects and are asked to make judgments about the similarities between each item and the others in the set. The task is to arrange the stimuli so that similar objects are positioned closer together and dissimilar objects are positioned further apart, such that the physical distance between the objects reflects their dissimilarity. Importantly, the criterion used to differentiate the items is chosen freely by the observer prior to sorting. The resulting physical distances between the objects can be transformed into a dissimilarity matrix which serves as a measure of the representational structure of the objects in the set, thus revealing the stimulus properties that are used to sort the items. Numerous studies have shown that the dissimilarity matrix obtained using inverse MDS corresponds to the underlying neural representations^25,26,30,33,34^. The dissimilarity matrix obtained using this method can also be used to test different theoretical models^26,31^.

Using inverse MDS in a between-subjects design, we compared the dissimilarity matrices obtained from a sorting task for stimuli that were displayed as scaled 2-D images, 3-D AR projections, or real solids. We were particularly interested in the extent to which physical and conceptual attributes of the items would contribute to sorting behavior in each display format. To identify these attributes, we examined the freely chosen criteria that participants used to arrange the stimuli. If the characteristics that observers use to distinguish between objects in a set remain stable across formats^9,23–26^, then similar sorting criteria should emerge from inverse MDS for 2-D images, 3-D AR projections and solids. Alternatively, if observers rely on different characteristics to distinguish between objects in different display formats, then different sorting criteria should emerge from inverse MDS. We hypothesized that, because 2-D images are abstractions (of real objects), they would be arranged according to abstract conceptual properties rather than physical properties. Because real objects (like 2-D images) obey statistical regularities regarding typical location, yet they convey richer information than 2-D images do about physical attributes such as size and weight, we predicted that they would be arranged according to both conceptual and physical properties. The question of whether sorting of AR stimuli would be similar or different than real objects is intriguing because, while AR stimuli are similar to real objects in their visual appearance and potential for interaction, they differ from real objects physically because they have no mass, and conceptually because they are not constrained by typical location.

## Results

**Figure 1:**
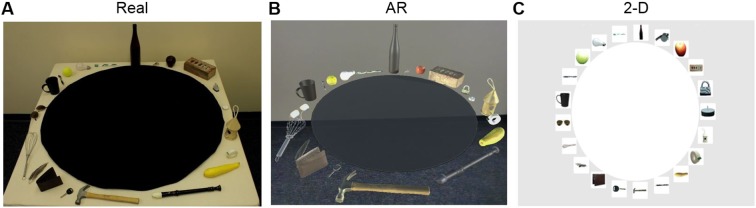
Observers viewed twenty-one different objects in one of three display formats. (**A**) In the real object condition, solid objects were displayed around a circular arena on a table. (**B**) In the Augmented Reality (AR) condition, ‘graspable’ 3-D holograms were displayed around a virtual black arena. (**C**) In the 2-D image condition, size-scaled images were displayed around a white arena on a computer monitor.

We used inverse multidimensional scaling^26,31^ to examine whether mental object representations are invariant to changes in display format. Two-hundred and sixty-four healthy observers were asked to position twenty-one different objects within an arena so that the distances between the items reflected their similarities and differences (Fig. 1). Prior to performing the sorting task, observers chose a criterion that best characterized how the objects differed from one-another. Critically, we used a between-subjects design in which the stimuli were presented to each observer in one of three display formats. Participants in the *real object* condition viewed real-world solid objects and during sorting they positioned the items manually upon a circular tabletop. Participants in the *AR* condition viewed high-fidelity 3-D computerized images of the objects via a Meta AR HMD. The AR stimuli were matched closely to the real objects for apparent distance, size, and background. Participants arranged the AR stimuli manually upon a ‘virtual’ circular table by reaching towards the object and moving it with the hand (see Method). Participants in the *2-D image* condition viewed planar colored images of the objects on an LCD computer monitor. Participants arranged the 2-D images within the circular arena on the screen using a drag-and-drop action with a computer mouse. As in previous studies of image vision (e.g.^25–28,35^), the 2-D images were scaled so that they had the same visual size.

One important difference between our experiment and previous studies that have used inverse MDS is that rather than selecting items that fall into clear categorical groups such as faces, bodies or animals^26,28,30^, the items in our set were highly heterogeneous. Our stimuli and design therefore permit a unique exploration of the characteristics that observers use to define and differentiate between objects in the absence of cues that would otherwise bias sorting criteria. The groupings that emerge from sorting provide powerful insights into the extent to which display format influences object responses when identity remains constant. Because the results of this study necessarily reflect the characteristics of the objects within the sample, future studies will be required to test whether similar sorting criteria are used to group objects of other types or categories.

### Declared sorting criteria

**Figure 2:**
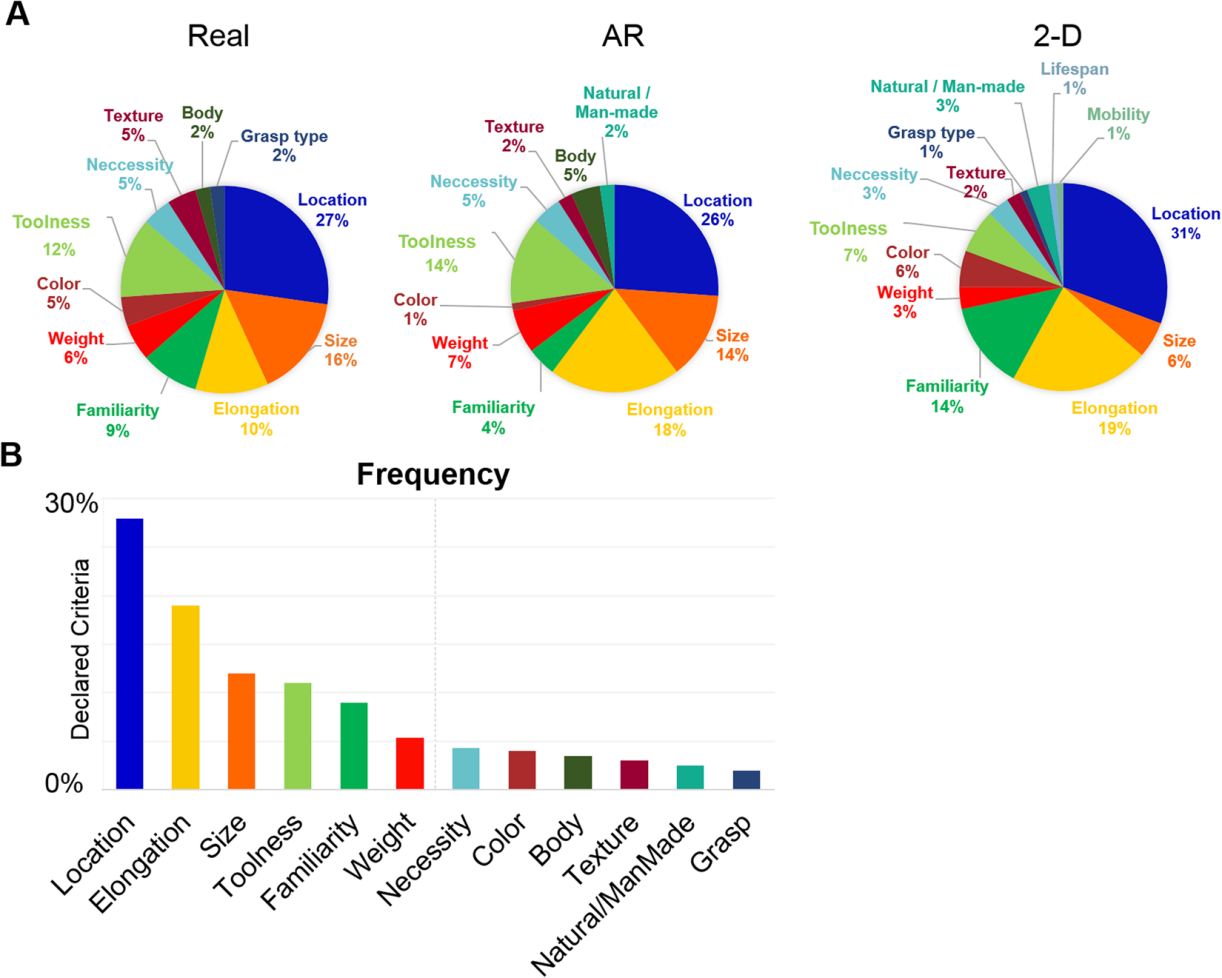
Frequency of declared object sorting criteria in each display format. (**A**) Declared sorting criteria for real objects (left), AR stimuli (middle) and 2-D images (right). (**B**) Frequency of sorting criteria across all display formats. Criteria to the left of the vertical dashed line were declared most frequently prior to sorting; these criteria were used to create the theoretical models. Infrequently selected or subjective criteria (right of dashed line) were not included in subsequent analyses.

Prior to initiating the manual sorting task, participants chose a sorting criterion that was applicable to all objects and verbally declared their criterion to the experimenter. Across all three display formats, typical location was selected most frequently as a sorting criterion (28%), followed by elongation (i.e. whether the object was compact or not) (19%), size (12%), toolness (i.e. whether or not the object is normally used for a specific purpose) (11%), familiarity (9%), and weight (5%) (Fig. 2). Although most criteria were selected by a percentage of observers in all display formats, some criteria were identified only in particular display formats. Specifically, for real objects (but not AR or 2-D images), a small percentage (2%) of observers proposed to sort the items according to how they would be grasped. Similarly, for real and AR objects (but not 2-D images) some observers (3.5%) proposed to sort the items according to whether or not the object was typically used on the body.

### Behavioral results

**Figure 3:**
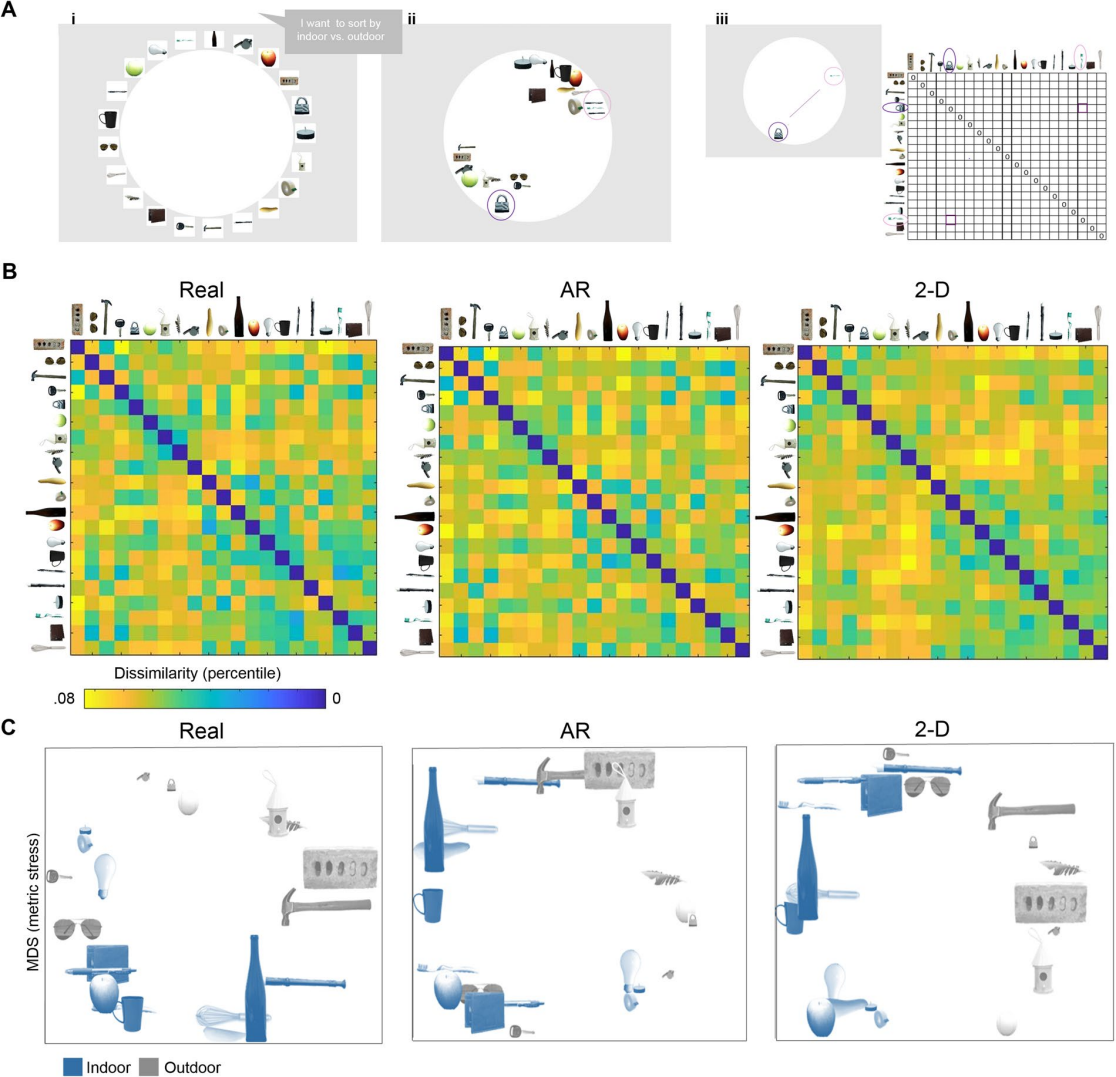
Methods to generate RDMs and model-free visualizations of the characteristics that were used to differentiate between objects during free sorting, separately for stimuli displayed as 2-D images, AR projections or real-world solids. (**A**) (i) Participants viewed the stimuli and then declared verbally a sorting criterion. The example depicts the 2-D image condition. (ii) Participants sorted the stimuli based on their proposed criterion. In this example, the observer chose to sort the stimuli according to the criterion of whether each item is typically found indoors vs. outdoors. (iii) The physical distance between sorted items reflects the perceived distance relative to the proposed criterion. The distances between items in the arena are transformed in Euclidean distance in the representational dissimilarity matrix (RDM): the father apart the items are in the arena, the higher their dissimilarity value in the RDM. (**B**) Average representational dissimilarity matrices (RDMs) generated based on sorting behavior for real objects (left), AR stimuli (middle), and 2-D images (right). Stimuli positioned closer together during sorting yield smaller numerical values in the RDM (i.e., low dissimilarity, illustrated by cooler colors); items positioned farther apart yield higher numerical values (i.e., high dissimilarity, illustrated by warmer colors). (**C**) Multidimensional scaling (MDS) plots for real objects (left), AR stimuli (middle), and 2-D images (right). For visualization purposes, hue differences in the plots denote typical location: indoor (blue) vs. outdoor (gray); object size represents relative differences in real-world size (larger image = larger real-world size).

Participants arranged items in the arena according to their declared criterion: the closer items were positioned in the arena, the more similar they were perceived by the participant with respect to the sorting criterion (Fig. 3A). From this spatial arrangement we inferred the dissimilarity structure, separately for each participant, in the form of a representational dissimilarity matrix (RDM) using inverse multidimensional scaling. Each RDM reflects the pairwise distances between the sorted items such that stimuli positioned closer together yield smaller numerical values (i.e., low dissimilarity) and those farther apart yield higher numerical values (i.e., high dissimilarity). Figure 3B shows the RDMs averaged across participants (RDM_data_), separately for each display format. The corresponding MDS plots for each display format are illustrated in Fig. 3C. The MDS plots permit a model-free visualization of the information contained in the RDMs: the farther apart two icons are in the MDS plot, the more dissimilar the items are in the corresponding RDM. For visualization purposes, we color-coded different items in the MDS plots in Fig. 3C according to the most frequently declared criterion, typical location (i.e. a conceptual property: indoor (blue) vs. outdoor (gray)). The sizes of the items in Fig. 3C represent the different real-world sizes of the objects (i.e., a physical property).

The apparent groupings of objects in multidimensional space reveal a number of interesting similarities and differences in the representations across display formats. First, the MDS plot for 2-D images shows a clear arrangement of the objects according to typical location, as is apparent in the corresponding RDM. For example, 2-D images of indoor objects, such as lightbulb, candle and tape, were positioned separately from outdoor objects, such as ball, birdhouse and whistle. Although a similar grouping according to typical location was evident for real objects, there were several inconsistencies in the case of the AR stimuli, for which the corresponding indoor items were positioned in proximity to outdoor items, and others were overlapping (i.e., hammer, recorder) in representational space. Second, real objects and AR stimuli, but not 2-D images, showed a clear grouping according to physical size. Objects that are typically larger in the real-world were grouped together and separately from items that are typically smaller in the real-world. For example, for real objects and AR stimuli, larger items such as hammer, brick, bottle and whisk were mostly grouped together and away from smaller objects, such as padlock and whistle. Conversely, for 2-D images, larger objects such as the hammer and brick were grouped in close proximity to smaller items such as the padlock and whistle, and away from other larger items such as whisk and bottle. Third, there were notable differences between the objects in our stimulus set with respect to mass (i.e., brick and hammer vs. feather), and these differences emerged in the MDS arrangements for real objects and AR stimuli. Specifically, heavier items were positioned together and separately from lighter exemplars. However, for the 2-D images, heavy and light items were proximal in the MDS arrangements. For example, the 2-D MDS shows the brick positioned closer to the feather than to the hammer.

### Correlations between theoretical models and sorting behavior

**Figure 4:**
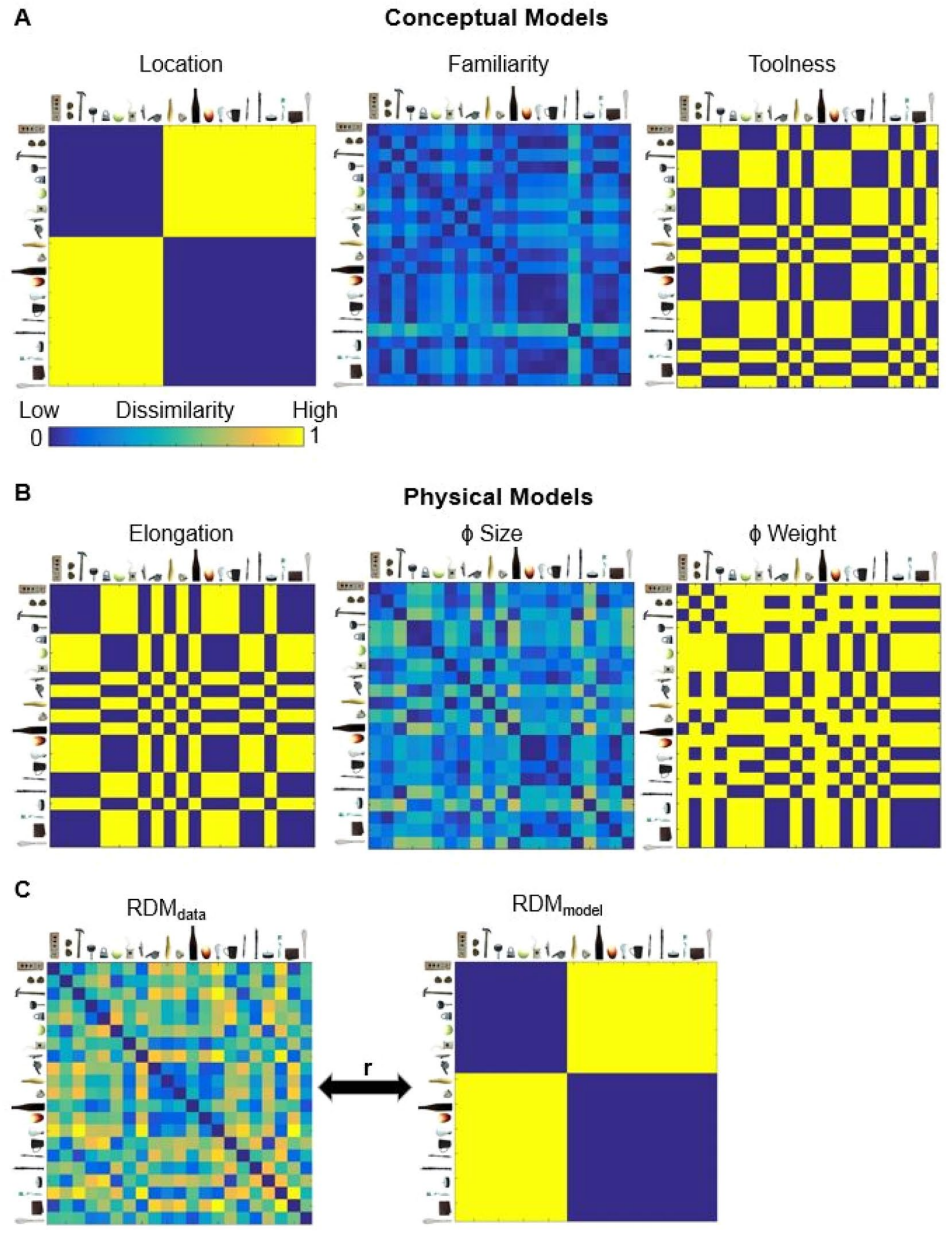
Theoretical models based on the declared object sorting criteria were correlated with the behavioral sorting data, separately for each display format. (**A**) Conceptual models. (**B**) Physical models. (**C**) Each participant’s RDM was correlated, using Spearman’s correlation coefficient, with the six different theoretical models (location model in the example).

Next, we measured and contrasted across display formats the extent to which different criteria were used to position the objects in the sorting task. We defined a set of conceptual and physical theoretical models based on participants’ declared sorting criteria, with the goal of measuring how much of the variance in the sorting behavior was explained by each theoretical model. To create the theoretical models, we excluded declared sorting criteria that were infrequent (<5% of respondents), did not arise in all three formats (i.e., ‘grasp type’, ‘bodyness’), or could not be generalized across participants (i.e., ‘necessity’; 4%) (Fig. 2B). For the six remaining criteria, three reflected conceptual object characteristics (location, toolness, familiarity) and three reflected physical object characteristics (elongation, size, weight). We generated a representational dissimilarity matrix model (RDM_model_) for the six criteria, each of which was based on ideal observer performance (see Fig. 4A,B**;** see Methods).

**Figure 5:**
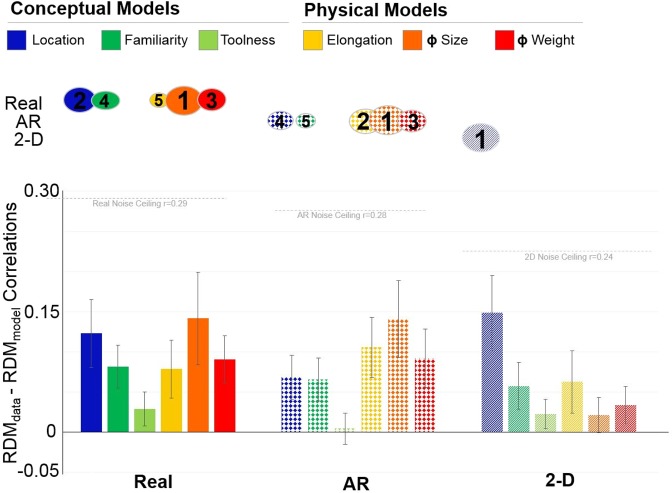
Mean RDM_data_ – RDM_model_ correlations for each display format. Lower panel: RDM_data_ - RDM_model_ correlations are plotted separately for each display format (real objects: solid fill; AR stimuli: cross-hatched fill; 2-D images: striped fill). Conceptual models are represented in cool colors (location: blue, familiarity: teal, toolness: green); physical models are represented by warm colors (elongation: yellow, real-world size: orange, weight: red). The noise ceiling is displayed, separately for each display format (grey dashed lines). The noise ceiling indicates the best performance that the models can reach based on the variability across participants. Upper panel: Ovals denote models in each display format that explained a significant amount of variance in stepwise linear regression analysis. The size of the ovals and the numbers inside the ovals reflect the importance of the models as predictors of object sorting behavior from the linear regression. Error bars represent +/− 95% CI mean.

Next, Spearman correlations between the behavioral RDM (RDM_data_) and the RDM of the model (RDM_model_) were calculated separately for each participant to obtain an RDM_data_-RDM_model_ correlation (Fig. 4C). The resulting correlations were averaged across participants (mean RDM_data_-RDM_model_) thus yielding a measure of the extent to which each theoretical model explained sorting behavior across observers (Fig. 5). *S*ingle-sample t-tests confirmed that mean RDM_data_-RDM_model_ correlations were significantly different from zero in all conditions (all p-values < 0.05), except for the Toolness model in the AR condition (t(87) = 0.451, p = 0.653, d = 0.048) and the Size model in the 2-D image condition (t(87) = 1.903, p = 0.060, d = 0.20), thus confirming that the models (derived from the verbal responses) captured the relevant variance.

To test whether the sorting criteria differed between formats, we compared mean RDM_data_ - RDM_model_ correlations across Display Formats (real, AR, 2-D) and Models (location, familiarity, toolness, elongation, size, weight) using 2-way mixed-model Analysis of Variance (ANOVA). A significant main effect of Display Format (F(1,2) = 8.67, p < 0.001, η_p_^2^ = 0.062) and Model (F(5,257) = 17.70, p < 0.001, η_p_^2^ = 0.256) was qualified by a significant two-way Display Format × Model interaction (F(10,514) = 2.548, p = 0.005 η_p_^2^ = 0.048), confirming that different representational structures emerged across formats.

One-way repeated measures ANOVAs comparing RDM_data_-RDM_model_ correlations separately for each display format revealed significant differences in the relative performance of the models for 2-D images (F(5,435) = 6.915, p < 0.001, η_p_^2^ = 0.088), AR stimuli (F(5,435) = 6.915, p < 0.001, η_p_^2^ = 0.074), and real objects (F(5,449) = 4.068, p = 0.009, η_p_^2^ = 0.045) and the pattern of effects was broadly consistent with the model-free organization of the data using MDS. For 2-D images, the conceptual location model (illustrated in blue in Fig. 5) performed significantly better than all other models (all p-values < 0.05). This result was reflected in the MDS visualizations (Fig. 3C, right panel) where indoor items showed a strong similarity as reflected by grouping together, and separately from outdoor items. Although the familiarity model performed marginally better than the toolness model (p = 0.048), there were no other differences in model performance for 2-D images (all p-values > 0.05). Conversely, for AR stimuli, the physical size model (illustrated in orange in Fig. 5) performed significantly better than all of the conceptual models (location, familiarity and toolness, all p-values > 0.05), but not significantly better than the physical weight (t = 1.612, p = 0.109, d = 0.24) or elongation (t = 1.151, p = 0.251, d = 0.174) models. The relevance of physical size and weight for AR stimuli was similarly reflected in the MDS plots in Fig. 3C, where items with similar size and weight were grouped more closely than items of different size and weight. The conceptual toolness model also performed worse than the other conceptual and physical models (all p-values < 0.05) for the AR stimuli. In contrast to 2-D images and AR stimuli for which correlations were highest for either conceptual or physical models respectively, for real objects a more multidimensional representational structure emerged in which the conceptual and physical models performed equally well. Although the toolness model performed worse than the other models for real objects (all p-values < 0.05), there were no other significant differences across models (all p-values > 0.05). These results are in line with the MDS visualizations (Fig. 3C, left panel), showing that real objects were grouped according to the conceptual property of location as well as the physical properties of size and mass.

Critically, contrasting RDM_data_ - RDM_model_ model performance across the display formats, pairwise comparisons revealed that the conceptual location model performed significantly worse for AR stimuli than for 2-D images (t(87) = 2.981, p = 0.003, d = 0.451) and real objects (t(87) = 2.170, p = 0.031, d = 0.327). Conversely, the physical size and weight models performed significantly worse for 2-D images than for real objects (size real objects vs. 2-D images: t(87) = 3.865, p < 0.001, d = 0.586; weight real objects vs. 2-D images: t(87) = 3.013, p = 0.003, d = 0.45), and AR stimuli (size AR stimuli vs. 2-D images: t(87) = 4.504, p < 0.001, d = 0.672; weight AR stimuli vs. 2-D images: t(87) = 2.698, p = 0.008, d = 0.403). There were no other differences in model performance across formats (all p-values > 0.05).

Finally, we used stepwise linear regression to evaluate whether the overall pattern of model contributions differed across display formats. For 2-D images, the overall model fit was R^2^ = 0.044. The conceptual location model was the only significant predictor of sorting behavior (Beta = 0.209, p < 0.001) (Fig. 5, upper panel). For AR stimuli, the overall model fit was R^2^ = 0.067; five of the six models were significant predictors of sorting behavior. The strongest predictor of object groupings for AR stimuli were the physical size (Beta = 0.215, p < 0.001), elongation (Beta = 0.149, p < 0.001) and weight (Beta = 0.124, p < 0.001) models. Following from the physical models the conceptual location (Beta = 0.079, p = 0.010) and familiarity (Beta = 0.074, p = 0.016) models. For real objects the overall model fit was R^2^ = 0.054. Five of the six models were significant predictors of sorting behavior. The strongest predictor of object groupings for real objects was the physical size model (Beta = 0.184, p < 0.001), followed by the conceptual location (Beta = 0.152, p < 0.001), physical weight (Beta = 0.096, p = 0.002), conceptual familiarity (Beta = 0.081, p = 0.009) and physical elongation (Beta = 0.076, p = 0.014) models, respectively.

## Discussion

Here, in a major departure from previous approaches that have studied stimuli in the form of 2-D computerized images of objects^25,26,30,31,33,34^, we used inverse MDS to investigate whether object responses are modulated by the format in which stimuli are displayed: 2-D computerized images, 3-D AR projections or real-world solids. Observers performed a sorting task using stimuli that were presented in one of the three different formats. Objects in the set were arranged so that the pairwise distances reflected the objects’ similarities and differences. We used MDS to isolate the characteristics of objects that were most salient in each format by correlating behavioral dissimilarity matrices derived from object sorting, with theoretical models that reflected a range of conceptual and physical object properties. If object processing remains stable across changes in display format, then similar groupings of objects should emerge from MDS across formats. On the contrary, however, we observed striking differences in the relative groupings of objects when the stimuli were displayed as 2-D images, AR projections, or as solids. Importantly, the results obtained from testing the theoretical models were in line with patterns that emerged from model-free organizations of the data using multidimensional scaling (Fig. 3C).

In line with the notion that pictures are abstract representations of real objects, we found that 2-D images of objects were coded in a unidimensional conceptual framework that reflected the typical location of the objects. The 2-D images in our paradigm, as in previous studies of object vision (e.g.^25–28,35^), were high-resolution colored photographs of everyday objects with equal retinal extent. The location model performed better than all other conceptual and physical models and was the only model that explained a significant amount of variance in the 2-D image groupings. These findings derived from inverse MDS support recent visual search and eye movement studies of 2-D images showing that humans are uniquely sensitive to the statistical relationships between pictured objects and their typical locations in everyday visual environments^29,36–42^. Although the criteria that we identified summarize the characteristics of the objects that comprised our stimulus set, our results bear a strong relationship with 2-D object properties that have been investigated in other studies of image vision^24,35,43–49^. For example, 2-D images have previously been shown to be represented in brain areas responsible for object recognition based on abstract conceptual properties such as location (where the object is typically found) and action (how the object is typically used)^45^.

Unlike the abstract conceptual structure that was apparent in responses to 2-D images, objects depicted as 3-D projections using AR elicited responses that reflected physical, rather than conceptual, properties such as real-world size and elongation. This qualitative shift in the nature of the responses from conceptual to physical attributes for 2-D and AR stimuli, respectively, is surprising given that previous studies have frequently reported comparable brain responses across 2-D to 3-D transitions^22,50,51^. Importantly, however, our AR stimuli depicted realistic everyday objects. Previous studies supporting 3-D shape invariance have typically used basic geometric shapes that lack meaning and familiar size associations^51^, or cues to surface material^52^. Our AR stimuli also conveyed rich stereoscopic information about 3-D volumetric structure and egocentric distance. Conversely, in previous studies, 3-D structure has been conveyed using monocular cues such as shading and specular highlights that do not reveal the distance of the object^51,52^, or via red-green anaglyphs for which zero order disparity cues give rise to the percept of planar contours that lie in front of or behind a fixation plane rather than as 3-D volumes^22^.

Another salient physical characteristic that emerged in the responses to AR stimuli was weight, as evinced by a strong correlation between the AR dissimilarity matrices and the weight model and strong performance of the weight model in stepwise linear regression. The influence of weight on behavioral responses to AR projections is intriguing because there are unambiguous visual (i.e., transparency) and top-down (i.e., wearing a HMD) cues that indicate to the observer that AR stimuli are digital projections that have no mass. These findings suggest that when a computerized stimulus conveys visual attributes that are consistent with real-world solids (together, probably, with information about surface material^53^) then attributes of solids that are not present in the stimulus are nevertheless incorporated into the behavioral responses. Future studies may determine whether the inclusion of weight as a relevant attribute of AR stimuli reflects anticipated physical characteristics of the stimulus (e.g., that the object would require motoric effort to lift or use) or more abstract semantic associations (i.e., that dense objects are usually heavy)^54^.

Unlike both 2-D images and AR stimuli, we found that real-world solids were processed using a rich multidimensional framework that incorporated both conceptual and physical object attributes. For real objects, the conceptual location model outperformed that of AR stimuli, the physical size and weight models outperformed that of 2-D images, and real objects were the only display type for which both conceptual and physical theoretical models performed equally well in direct contrasts of RDM-model correlations. Similarly, stepwise regression revealed a unique balance in the contribution of conceptual and physical models in explaining overall variance in the real object arrangements. These findings from behavioral inverse MDS demonstrate that real objects are processed, in part, according to conceptual characteristics, thus confirming and extending previous results from image vision which have shown that contextual information in 2-D scenes can facilitate the recognition of objects in the scene^2,29^. The relevance of conceptual information about location for both real and 2-D objects is consistent with the idea that we frequently encounter and use real objects in specific locations which could elicit powerful expectations about co-occurrence^55^. At the same time, our results from inverse MDS complement and critically extend emerging data from behavioral^56^, fMRI^57,58^, EEG^59^ and neuropsychological studies^13,60,61^, which have highlighted quantitative and qualitative differences in the way real objects and computerized images are processed during perception^13^, memory^62^, attention^63^ and decision-making^64,65^. For example, real objects have been shown to elicit little if any fMRI repetition suppression (fMRI-RS), unlike 2-D images of the same objects, for which fMRI-RS effects are widespread throughout ventral and dorsal cortex^57^. Evidence from high-density EEG indicates that real objects trigger stronger and more prolonged automatic motor preparation signals than do matched images of the same objects, particularly in the hemisphere contralateral to the dominant hand^59^. The data reported here advance this work by revealing which characteristics of real objects are most powerful in driving the reported behavioral responses. Future neuroimaging studies will determine whether differences we have revealed in the nature of object coding across display formats using behavioral MDS, are similarly evident in cortical representations derived from neural measures.

Here, we demonstrate that differences in the visual appearance, but not the identity, of objects influences behavioral responses. Our results show that scaled 2-D images, like those used in most studies of object perception, elicit responses that are fundamentally different to AR projections and real-world solid exemplars of the same objects. Our findings provide a striking demonstration that changes in the appearance of an object, but not its identity, can lead to shifts in the associated responses. Relying on scaled 2-D images of objects appears to limit the basic characteristics that observers derive during object responses; this does not appear to be the case for other types of stimuli including AR projections and real objects. This stimulus-dependent shift in the underlying nature of object processing could have remained elusive in previous studies (such as those that have manipulated stimulus characteristics such as size and display format) because the investigations have been limited to the types of object characteristics that can be conveyed using pictorial cues.

Our results drawn from an object sorting task lend support previous studies that have reported size invariance in picture perception. Invariance in behavioral responses to 2-D picture of objects has been demonstrated across a range of behavioral tasks, including object recognition^13^, priming^7^, perceptual learning^15^ and visual search^16^, as well as across a wide range of 2-D picture formats, from line-drawings to greyscale shaded photographs. Retinal size may be discounted during 2-D object processing because image size conveys no information about the real-world size of the stimulus. For example, in a recent study, Holler *et al*.^13^ tested object recognition in neuropsychological patients with visual agnosia due to bilateral lesions of ventral cortex. As expected, the patients were severely impaired in their ability to recognize 2-D images of objects. However, the patients showed a striking preservation in their ability to recognize everyday real-world objects. Importantly, the recognition advantage for real objects was only apparent when the physical size of the object matched its typical real-world size. Recognition of real objects whose physical size deviated above or below real-world size was severely impaired. Analogous manipulations of the retinal size of 2-D photographs of the same objects (or of basic geometric shapes that have no real-world size association) had no influence on recognition^13^.

Nevertheless, despite convergent evidence in previous studies for size invariance in picture perception, across a range of measures, tasks, and stimulus formats, using stimuli that varied twofold (or more) in size, our results leave open the question of whether observers might rely more on size during object sorting if the visual size of 2-D images were consistent with the real-world size. Previous studies have frequently used stimuli that are equated for retinal size because this was a visual dimension of the stimuli that was controlled for, akin to presenting grayscale images to avoid color-related signals. Accordingly, we scaled our 2-D images so that we could compare object responses between stimuli similar in appearance to those used in laboratory studies of object vision versus the types of objects encountered in real-world scenarios. In our study, the presence or absence of a given attribute in the stimulus did not guarantee that the attribute was evident in (or absent from) the object responses. For example, although the 2-D images conveyed information about elongation, elongation was not a significant predictor of the 2-D object groupings. Similarly, although weight was not physically present in the AR stimuli, weight had a surprisingly powerful influence on the AR object groupings. Recent fMRI and behavioral findings show that object responses can be influenced by mid-level stimulus features such as ‘boxiness’ or ‘curviness’ that presumably convey the likelihood that the depicted object is large or small in the real-world^24,35,47,48^. Our results suggest that while object processing may be modulated by mid-level stimulus features, the presence of concrete size information has a relatively more powerful effect on responses. Future research will be required to delineate the extent and limits of invariance in 2-D image processing. Important avenues for follow-up investigation will be to examine whether observers rely on size cues for 2-D images of objects whose retinal size matches the real-world size (for example, if they were displayed using a projector) during object sorting, as well as in other tasks including recognition, priming, perceptual learning and visual search. Our emphasis, however, is on the major finding that scaled 2-D images constrain the characteristics of object responses, whereas other, richer stimulus formats do not.

The differences we observed in object responses for 2-D images, AR stimuli and real objects could also reflect differences in the action affordances of the stimuli. Whereas real objects and AR stimuli afford grasping and manipulation, 2-D images do not. Previous studies that have directly compared grasping movements towards objects in different display formats have reported behavioral^66–69^ and neural differences^58^ between real objects and 2-D images. For example, Holmes and Heath^67^ found that grasping is differently influenced by visual information for 2-D and 3-D objects. In an fMRI study, Freud and colleagues^58^ measured dissociable neural representations for 2-D images and real objects in the key grasping area, anterior intraparietal sulcus. Differences in the observer’s task^34,70,71^ or behavioral goals^72,73^ have been shown to alter object representations across cortex. For example, decoding object identity from fMRI responses in ventral-temporal and frontal cortices is less accurate when an observer’s task changes from focusing on conceptual properties (i.e., man-made vs. natural object) to physical properties (i.e., object color)^74^. The idea that object processing may be influenced by action affordances is further supported by recent evidence from behavioral psychophysics, which demonstrates that real objects capture attention more so than 2-D or 3-D stereoscopic images, but only when the (real) objects are within reach^63^. These unique effects of real objects on attention disappear when the stimuli are positioned out of reach, or behind a transparent barrier that prevents in-the-moment interaction with the stimuli^63^.

The differences we observed in object responses for 2-D images, AR stimuli and real objects could reflect differences in the nature of the task performed on the stimuli. Specifically, participants in the current study who sorted 2-D images used a drag-and-drop action with a computer mouse, while those who saw AR projections and real objects used a manual grasping action. However, although a similar manual task was used to sort both the real objects and AR stimuli, the object responses were different across both formats. Other studies have found that attention can modulate object representations in human cortex^75–80^. Although participants in our task may have attended to abstract (i.e., conceptual) versus concrete (i.e., physical) object features^81^ in the 2-D image versus real object and AR display formats, respectively, accounts that appeal to differences in attention must nevertheless explain why different stimulus formats selectively draw attention towards conceptual, physical, or multidimensional object properties.

In summary, our results suggest that real-world objects are processed in a richer, more multidimensional framework compared to computerized 2-D and AR image displays^13,62,63,65^. Real objects, unlike 2-D images and AR projections, provide critical information about object identity and physical characteristics, and we show that these conceptual and physical properties are also evident in behavioral responses. Future studies will be necessary to disentangle the extent to which different visual stimulus characteristics (*such as visual size*), action affordances, and response tasks, influence object processing. Although visual processing in the service of object recognition is thought to require that shape information is extracted independently of the visual cues that define the shape^3,20,22,51,82–84^, our results suggest that there may be a qualitative shift in object responses when richer, more naturalistic, stimuli are used. Although regions of posterior parietal cortex are known to be sensitive to the 3-D shape of object images^85–89^, much of the dorsal visual pathway is dedicated to processing physical object properties, such as real-world size^90–92^ and weight^93^, in the service of goal-directed actions^94^. These findings raise fundamental questions about the extent to which scaled computerized images characterize the multidimensional nature of object processing in naturalistic environments, and highlight the need for more comprehensive theoretical accounts of object vision^1,43,81^.

## Method

### Participants

Two hundred and sixty-four healthy adult volunteers (mean age 20 years, 196 females, 246 right handers) participated in the study for course credit (88 participants per display format). Observers were required to have normal to corrected normal vision and to be able to provide informed consent. All experimental procedures were approved by the University of Nevada Internal Review Board and Ethics Committee. All methods were performed in accordance with the relevant guidelines and regulations. Informed consent was obtained from all participants that participated in the study.

### Stimuli and apparatus

#### Real Objects

The stimulus set was comprised of twenty-one different everyday objects: apple, bird house, brick, car key, coffee cup, feather, hammer, lightbulb, padlock, pen, recorder, sunglasses, scotch tape roll, tea-light candle, tennis ball, toothbrush, yellow squash, wallet, whisk, wine bottle, and whistle. The stimuli spanned a variety of conceptual (location, toolness) and physical properties (shape, size, weight, color, surface material). The real objects were displayed around a 42 × 42in black circular arena, on top of a 51.5 × 51.5 inch table (Fig. 1A).

#### 3-D Augmented Reality (AR)

To create the AR stimuli, the twenty-one real objects were 3-D scanned and edited using an Artec Spider 3D scanner and software (version 12, Professional Edition, Artec3d.com). The glasses and the whisk could not be 3-D scanned due to the shiny surface (glasses) and thin lines (whisk); similar 3-D renderings of these objects were sourced from an online database (GrabCAD.com) and the images were digitally modified using Solidworks (Solidworks.com) to match the real object stimuli. The AR stimuli were displayed using Unity software (version 2017.3.Of3 Personal, Unity.com). 3-D renderings of the objects were placed around a black circular arena (sized to be 42 × 42 inches based on a 55.5 IPD) so that they appeared to be displayed on a table top analogous to that used in the real object condition (Fig. 1B). Using the Meta 2 headset hand recognition algorithms (metavision.com), participants were able to interact with the AR stimuli using relatively naturalistic reaching movements.

#### 2-D Images

High resolution photographs of the twenty-one real objects were taken using a Canon Rebel T2i DSLR camera with constant F-stop and shutter speed. The photos were edited to remove background and resized to fit within a 600 × 600 pixel square. Thus, similar to previous behavioral and fMRI studies investigating object processing (e.g.^24,26,31,35^, the 2-D images had the same physical size on the screen (Fig. 1C). The 2-D images were displayed on a 27 inch ACER G27HL LCD monitor from a Dell Latitude E6430 computer and Logitech K120 keyboard and mouse.

### Procedure

#### Inverse multi-dimensional scaling

We used the inverse multi-arrangement scaling method in a between-subjects design to infer pairwise dissimilarities from 2-D arrangements of items^26,31^. Participants were initially presented with the items (in one display format) and instructed as follows: “arrange the objects according to their similarity”. Following from Mur *et al*.^26^, the instructions did not specify a specific sorting criterion. Participants were asked not to interact with the stimuli prior to making a decision about their chosen sorting criterion. Participants were instructed to choose any criterion, as long as it was possible to sort all the stimuli along that dimension, and they were given unlimited time to choose the criterion. We asked participants to declare the sorting criterion in order to create models based on those criteria, rather than on a-priori hypotheses, although this may increase the risk of introducing a demand characteristic. After participants indicated verbally their proposed sorting criterion to the experimenter, they were asked to reiterate the instructions to demonstrate comprehension of the sorting task. Finally, participants were asked to sort the objects manually so that the physical distance between objects reflected the degree of dissimilarity. After sorting, a representational dissimilarity matrix (RDM) was generated using the Matlab (mathworks.com) code provided by Mur and used in previous studies^26,31^. Specifically, the physical distances between each pair of items in the arena was transformed in a dissimilarity estimate using Euclidean distances, as showed in Fig. 3A. Because we aimed to compare broad categorization patterns across different display formats, rather than multidimensionality within display formats, we asked participants to sort the objects only once, instead of multiple times as in previous studies^26,31^. After the sorting the objects was completed, we collected further ratings for the objects. We measured familiarity by asking participants to rate how often they encountered each object, on a scale from 1 (never), 2 (yearly), 3 (monthly), 4 (weekly) and 5 (daily). Participants completed the rating task while viewing the objects alphabetically in the display format used in the main experiment. For the real and AR conditions, participants gave a verbal response that was recorded by the experimenter. For the 2-D image condition, participants manually entered their ratings using a keyboard.

Real objects. To complete the sorting task, participants manually arranged the objects on the circular arena. Participants were not allowed to touch or interact with the items before deciding on their chosen criterion. After sorting was complete, each participant’s sorting arrangement was photographed. Then, the spatial arrangement of the items on the table was recreated by the experimenter on a computer screen, by dragging images of the items inside the white arena (Fig. 1**)** created using the Matlab code^26,31^ for inverse multidimensional scaling.

3-D Augmented reality. The AR head-mounted display (HMD) was calibrated separately for each participant to match ocular characteristics (interpupillary distance and eye alignment) using Meta software (SDK 2.7.0 Unity Package, metavision.com). Participants then mapped the headset to the environment, thereby allowing the visual display to be anchored to a point in space. Next, participants were familiarized with the AR headset by using the hands to move a sphere onto a circular arena. To move objects in the AR environment, participants opened the grasping hand (with fingers fully extended) in the area where the object appeared and then closed the hand over the object. Objects could be moved by closing the fingers, moved around using a closed fist, and subsequently dropped into position by extending the fingers. After the sorting task was completed, a screenshot of the sorting arrangement was captured, and (as in the real object condition) the arrangement was recreated on the white arena in the computer screen, as done for the real objects and 2-D images.

2-D images. Using Matlab code^26,31^, the 2-D images were displayed on a gray background around a white circular arena (Fig. 1C). After selecting a sorting strategy, participants used a drag-and-drop action with a computer mouse to arrange objects inside of the arena.

### Data analysis

Using G*Power^95^, we estimated that a total of 86 participants were required per display format to reveal a medium effect size with 90% power. The goal of the analysis was to measure the extent to which different theoretical models explained variance in object sorting behavior.

#### Declared sorting criteria and behavioral analyses

For each participant, the distances between objects in the arena were transformed into a representational dissimilarity matrix (RDM) using Matlab code previously used in other studies^26,31^. The Matlab code uses Euclidean distances to compute the level of dissimilarity between pairs of items based on the physical distance between these items in the arena. For example, if two objects are placed close together in the arena, their value of dissimilarity in the RDM is small. The resulting RDMs were then averaged across participants. The resulting representational dissimilarity matrix (RDM_data_) was then visualized as MDS plot, in which the distance between the icons reflects the degree of dissimilarity. The MDS plot was created using the Matlab function mdscale (criterion: metric stress). We examined the declared sorting criteria by visualizing the percentage of times different criteria were proposed. The data were visualized separately for each display format using pie charts (Fig. 2A); mean % of responses for each criterion were then averaged across display formats and visualized in Fig. 2B as a bar graph.

#### Correlations between theoretical models and sorting behavior

To create the theoretical models, we excluded declared sorting criteria that were very infrequent (<5% of respondents), did not arise in all three formats, or could not be generalized across participants. The resulting six most frequent sorting criteria served as theoretical models in the subsequent analyses. Three of the top six criteria focused on conceptual object properties and the remaining three criteria were focused on physical object properties. For the conceptual models: the location model reflected whether an object is typically found indoors or outdoors; the toolness model reflected whether or not an object is typically used for a specific purpose^96^; the familiarity model reflected the difference in the frequency judgments between pairs of items based on the result of the frequency of occurrence questionnaire. The average score for each object was calculated separately for participants in each display format group using the equation: absolute value (object A − object B)/(object A + object B). The resulting scores were averaged across participants within each display format. The correlations between the familiarity models obtained for the different display formats were high (r > 0.95 for all comparisons), indicating that the frequency judgments were almost identical across display formats. As a representative frequency model, the real object familiarity model is depicted in Fig. 4. The elongation model was created based on whether the objects were elongated (longer length than width) or compact (similar length and width). The size model was created using OnShape modeling software^97^ (OnShape.com) to build a box that would represent the absolute minimum size to fit each real object^47^. Using OnShape, we calculated the diagonal of each box for each stimulus. To find the relative pairwise dissimilarity between two objects we used the formula: absolute value (object A − object B)/(object A + object B). The weight model was created based on whether the objects were light (0.001 grams to 1.4 grams), medium weight (2.5 grams to 10 grams), or heavy (>10 grams).

Finally, the data for each participant (RDM_data_) was correlated (Spearman’s correlations) with the representational dissimilarity matrix of each theoretical model (RDM_model_) (Fig. 4C). The resulting RDM_data_-RDM_model_ correlations were averaged across participants, separately for each display format (Fig. 5). To measure the level of noise in the data, we calculated a noise ceiling that reflects the maximum correlation that a theoretical model can achieve given the variability between participants within each display format. To do this, we z-transformed each participant’s RDM_data_. Next, we used a leave-one-out approach, correlating each participants RDM_data_ (Spearman’s correlation coefficient) with the average RDM of the other participants (for a similar approach, see^25,98^). To test the extent to which the theoretical models explained the variance in the behavioral data, we conducted Analysis of Variance followed by single-sample t-tests, where appropriate. To evaluate whether the overall pattern of model contributions differed across display formats we conducted a stepwise linear regression separately for each display format. All data were checked for normality and all statistical tests were two-tailed.

## Data Availability

The data that support the findings of this study are available from the corresponding author on request. There are no restrictions on the sharing of the data, apart from allowing sufficient time to curate and send them on request.

## Code availability

Please see Kriegeskorte and Mur (2012)^31^ for the inverse MDS Matlab code which was used to assess dissimilarities for the experiment. The code used to analyze the data is available from the corresponding author upon reasonable request.

## References

[1] Grill-Spector K, Malach R (2004). The human visual cortex. Annu. Rev. Neurosci..

[2] Bar M (2004). Visual objects in context. Nat. Rev. Neurosci..

[3] Grill-Spector K, Kourtzi Z, Kanwisher N (2001). The lateral occipital complex and its role in object recognition. Vis. Res..

[4] Logothetis NK, Sheinberg DL (1996). Visual object recognition. Annu. Rev. Neurosci..

[5] Kravitz DJ, Vinson LD, Baker CI (2008). How position dependent is visual object recognition?. Trends Cogn. Sci..

[6] Biederman I, Cooper EE (1991). Evidence for complete translational and reflectional invariance in visual object priming. Perception.

[7] Fiser J, Biederman I (1995). Size invariance in visual object priming of gray-scale images. Perception.

[8] Wiggs CL, Martin A (1998). Properties and mechanisms of perceptual priming. Curr. Opin. Neurobiol..

[9] Vuilleumier P, Henson RN, Driver J, Dolan RJ (2002). Multiple levels of visual object constancy revealed by event-related fMRI of repetition priming. Nat. Neurosci..

[10] Zimmer M, Kovacs G (2011). Position specificity of adaptation-related face aftereffects. Philos. Trans. R. Soc. Lond. B. Biol. Sci..

[11] Cox DD, Meier P, Oertelt N, DiCarlo JJ (2005). ‘Breaking’ position-invariant object recognition. Nat. Neurosci..

[12] Jolicoeur, P. A size-congruency effect in memory for visual shape. *Mem. Cognit*. **151**. **Jolic**, 531–543 (1987). 10.3758/bf03198388 3695948

[13] Holler DE, Behrmann M, Snow J (2019). Real-world size coding of solid objects, but not 2-D or 3-D images, in visual agnosia patients with bilateral ventral lesions. Cortex..

[14] Biederman, I. & Cooper, E. E. Size in variance in visual object priming. *J. Exp. Psychol. Hum. Percept. Perform*. vol. 18 (1992).

[15] Furmanski CS, Engel SA (2000). Perceptual learning in object recognition: object specificity and size invariance. Vision Res..

[16] Ahissar M, Hochstein S (1993). Attentional control of early perceptual learning. Proc. Natl. Acad. Sci. USA.

[17] Malach R (1995). Object-related activity revealed by functional magnetic resonance imaging in human occipital cortex. Proc. Natl. Acad. Sci..

[18] Grill-Spector K (1999). Differential processing of objects under various viewing conditions in the human lateral occipital complex. Neuron.

[19] Grill-Spector K, Kushnir T, Edelman S, Itzchak Y, Malach R (1998). Cue-invariant activation in object-related areas of the human occipital lobe. Neuron.

[20] Kourtzi Z, Erb M, Grodd W, Bulthoff HH (2003). Representation of the perceived 3-D object shape in the human lateral occipital complex. Cereb. Cortex (New York, NY).

[21] Nishimura M, Scherf KS, Zachariou V, Tarr MJ, Behrmann M (2015). Size precedes view: developmental emergence of invariant object representations in lateral occipital complex. J. Cogn. Neurosci..

[22] Kourtzi Z, Kanwisher N (2000). Cortical regions involved in perceiving object shape. J. Neurosci..

[23] Ishai A, Ungerleider LG, Martin A, Haxby JV (2000). The representation of objects in the human occipital and temporal cortex. J. Cogn. Neurosci..

[24] Konkle T, Oliva A (2012). A real-world size organization of object responses in occipitotemporal cortex. Neuron.

[25] Bracci S, Op de Beeck H (2016). Dissociations and associations between shape and category representations in the two visual pathways. J. Neurosci..

[26] Mur M (2013). Human object-similarity judgments reflect and transcend the primate-IT object representation. Front. Psychol..

[27] Kriegeskorte N (2008). Matching categorical object representations in inferior temporal cortex of man and monkey. Neuron.

[28] Cichy RM, Kriegeskorte N, Jozwik KM, van den Bosch JJF, Charest I (2019). The spatiotemporal neural dynamics underlying perceived similarity for real-world objects. Neuroimage.

[29] Oliva A, Torralba A (2007). The role of context in object recognition. Trends Cogn. Sci..

[30] Charest I, Kievit RA, Schmitz TW, Deca D, Kriegeskorte N (2014). Unique semantic space in the brain of each beholder predicts perceived similarity. Proc. Natl. Acad. Sci. USA.

[31] Kriegeskorte N, Mur M (2012). Inverse MDS: Inferring dissimilarity structure from multiple item arrangements. Front. Psychol..

[32] Gärdenfors, P. *Conceptual spaces*: *the geometry of thought*. (MIT Press, 2004).

[33] Bracci S, Caramazza A, Peelen MV (2015). Representational similarity of body parts in human occipitotemporal cortex. J. Neurosci..

[34] Bracci S, Daniels N, Op de Beeck H (2017). Task context overrules object- and category-related representational content in the human parietal cortex. Cereb. Cortex (New York, NY).

[35] Konkle T, Oliva A (2012). A familiar-size Stroop effect: real-world size is an automatic property of object representation. J. Exp. Psychol. Hum. Percept. Perform..

[36] Eckstein, M. P. Visual search: a retrospective. *J. Vis*. **11** (2011). 10.1167/11.5.14 22209816

[37] Neider MB, Zelinsky GJ (2006). Scene context guides eye movements during visual search. Vis. Res..

[38] Castelhano MS, Heaven C (2010). The relative contribution of scene context and target features to visual search in scenes. Atten. Percept. Psychophys..

[39] Mack SC, Eckstein MP (2011). Object co-occurrence serves as a contextual cue to guide and facilitate visual search in a natural viewing environment. J. Vis..

[40] Malcolm GL, Henderson JM (2010). Combining top-down processes to guide eye movements during real-world scene search. J. Vis..

[41] Wolfe JM, Alvarez GA, Rosenholtz R, Kuzmova YI, Sherman AM (2011). Visual search for arbitrary objects in real scenes. Atten. Percept. Psychophys..

[42] Vo ML, Henderson JM (2010). The time course of initial scene processing for eye movement guidance in natural scene search. J. Vis..

[43] Peelen MV, Kastner S (2011). Is that a bathtub in your kitchen?. Nat. Neurosci..

[44] Peelen MV, Kastner S (2014). Attention in the real world: toward understanding its neural basis. Trends Cogn. Sci..

[45] Peelen MV, Caramazza A (2012). Conceptual object representations in human anterior temporal cortex. J. Neurosci..

[46] Perini F, Caramazza A, Peelen MV (2014). Left occipitotemporal cortex contributes to the discrimination of tool-associated hand actions: fMRI and TMS evidence. Front. Hum. Neurosci..

[47] Konkle T, Oliva A (2011). Canonical visual size for real-world objects. J. Exp. Psychol. Hum. Percept. Perform..

[48] Long, B., Moher, M., Carey, S. & Konkle, T. Real-world size is automatically encoded in preschoolers’ object representations. *J. Exp. Psychol. Hum. Percept. Perform* (2018). 10.1037/xhp0000619 30985176

[49] Long B, Konkle T, Cohen MA, Alvarez GA (2016). Mid-level perceptual features distinguish objects of different real-world sizes. J. Exp. Psychol. Gen..

[50] Vinberg J, Grill-Spector K (2008). Representation of shapes, edges, and surfaces across multiple cues in the human visual cortex. J. Neurophysiol..

[51] Konen CS, Kastner S (2008). Two hierarchically organized neural systems for object information in human visual cortex. Nat. Neurosci..

[52] Eger E, Ashburner J, Haynes JD, Dolan RJ, Rees G (2008). fMRI activity patterns in human LOC carry information about object exemplars within category. J. Cogn. Neurosci..

[53] Cant JS, Goodale MA (2011). Scratching beneath the surface: new insights into the functional properties of the lateral occipital area and parahippocampal place area. J. Neurosci..

[54] Buckingham, G. Examining the size-weight illusion with visuo-haptic conflict in immersive virtual reality. *Q. J. Exp. Psychol. (Hove)*. 1747021819835808, 10.1177/1747021819835808 (2019). 10.1177/1747021819835808 30789088

[55] Galleguillos, C., Rabinovich, A. & Belongie, S. Object categorization using co-occurrence, location and appearance. in *26th IEEE Conference on Computer Vision and Pattern Recognition, CVPR*, 10.1109/CVPR.2008.4587799 (2008).

[56] Gerhard, T. M., Culham, J. C. & Schwarzer, G. Distinct Visual Processing of Real Objects and Pictures of Those Objects in 7- to 9-month-old Infants. *Frontiers in Psychology* **7**, 827 (2016). 10.3389/fpsyg.2016.00827 PMC4904016 27378962

[57] Snow JC (2011). Bringing the real world into the fMRI scanner: repetition effects for pictures versus real objects. Sci. Rep..

[58] Freud E (2018). Getting a grip on reality: Grasping movements directed to real objects and images rely on dissociable neural representations. Cortex..

[59] Marini, F., Breeding, K. A. & Snow, J. C. Distinct visuo-motor brain dynamics for real-world objects versus planar images. *Neuroimage*, 10.1016/j.neuroimage.2019.02.026 (2019). 10.1016/j.neuroimage.2019.02.026 PMC6536332 30776529

[60] Chainay, H. & Humphreys, G. W. The real-object advantage in agnosia: Evidence for a role of surface and depth information in object recognition. *Cognitive**Neuropsychology* **18**(2), 175–191 (2010). 10.1080/02643290042000062 20945210

[61] Humphrey, G. K., Goodale, M. A., Jakobson, L. S. & Servos, P. The role of surface information in object recognition: studies of a visual form agnosic and normal subjects. *Perception*, **23**(12), 1457–1481 (1994). 10.1068/p231457 7792135

[62] Snow JC, Skiba RM, Coleman TL, Berryhill ME (2014). Real-world objects are more memorable than photographs of objects. Front. Hum. Neurosci..

[63] Gomez, M. A., Skiba, R. M. & Snow, J. C. Graspable objects grab attention more than images do. *Psychol. Sci*. 956797617730599, 10.1177/0956797617730599 (2017). 10.1177/0956797617730599 PMC5809313 29215960

[64] Romero, C. A. & Snow, J. C. Methods for presenting real-world objects under controlled laboratory conditions. *J. Vis. Exp*., 10.1016/j.cortex.2017.11.010. 10.3791/59762 PMC7228680 31282889

[65] Romero, C. A., Compton, M. T., Yang, Y. & Snow, J. C. The real deal: Willingness-to-pay and satiety expectations are greater for real foods versus their images. *Cortex*, 10.1016/j.cortex.2017.11.010 (2017). 10.1016/j.cortex.2017.11.010 PMC5966320 29233524

[66] Ganel, T., Chajut, E. & Algom, D. Visual coding for action violates fundamental psychophysical principles. *Curr. Biol*. vol. 18 (2008). 10.1016/j.cub.2008.04.052 18644333

[67] Holmes SA, Heath M (2013). Goal-directed grasping: The dimensional properties of an object influence the nature of the visual information mediating aperture shaping. Brain Cogn..

[68] Ganel T, Goodale MA (2003). Visual control of action but not perception requires analytical processing of object shape. Nature.

[69] Chen J, Sperandio I, Goodale MA (2015). Differences in the effects of crowding on size perception and grip scaling in densely cluttered 3-D scenes. Psychol. Sci..

[70] Cavina-Pratesi C, Goodale MA, Culham JC (2007). FMRI reveals a dissociation between grasping and perceiving the size of real 3D objects. PLoS One.

[71] Vaziri-Pashkam M, Xu Y (2017). Goal-directed visual processing differentially impacts human ventral and dorsal visual representations. J. Neurosci..

[72] Gilbert CD, Li W (2013). Top-down influences on visual processing. Nat. Rev. Neurosci..

[73] Lamme VA, Roelfsema PR (2000). The distinct modes of vision offered by feedforward and recurrent processing. Trends Neurosci..

[74] Harel A, Kravitz DJ, Baker CI (2014). Task context impacts visual object processing differentially across the cortex. Proc. Natl. Acad. Sci. USA.

[75] Beauchamp MS, Cox RW, DeYoe EA (1997). Graded effects of spatial and featural attention on human area MT and associated motion processing areas. J. Neurophysiol..

[76] Chawla D, Rees G, Friston KJ (1999). The physiological basis of attentional modulation in extrastriate visual areas. Nat. Neurosci..

[77] Huk AC, Heeger DJ (2000). Task-related modulation of visual cortex. J. Neurophysiol..

[78] Runeson E, Boynton GM, Murray SO (2013). Effects of task and attentional selection on responses in human visual cortex. J. Neurophysiol..

[79] Beck DM, Kastner S (2005). Stimulus context modulates competition in human extrastriate cortex. Nat. Neurosci..

[80] Murray SO, Wojciulik E (2004). Attention increases neural selectivity in the human lateral occipital complex. Nat. Neurosci..

[81] Plaut DC, Farah MJ (1990). Visual object representation: Interpreting neurophysiological data within a computational framework. J. Cogn. Neurosci..

[82] Vogels R, Orban GA (1996). Coding of stimulus invariances by inferior temporal neurons. Prog. Brain Res..

[83] Sary G, Vogels R, Orban GA (1993). Cue-invariant shape selectivity of macaque inferior temporal neurons. Science..

[84] Tanaka H, Uka T, Yoshiyama K, Kato M, Fujita I (2001). Processing of shape defined by disparity in monkey inferior temporal cortex. J. Neurophysiol..

[85] Freud, E. *et al*. Three-dimensional representations of objects in dorsal cortex are dissociable from those in ventral cortex. *Cereb. Cortex*, 10.1093/cercor/bhv229 (2015). 10.1093/cercor/bhv229 PMC13100970 26483400

[86] Durand J-B, Peeters R, Norman JF, Todd JT, Orban GA (2009). Parietal regions processing visual 3D shape extracted from disparity. Neuroimage.

[87] Georgieva S, Peeters R, Kolster H, Todd JT, Orban GA (2009). The processing of three-dimensional shape from disparity in the human brain. J. Neurosci..

[88] Orban GA (2011). The Extraction of 3D shape in the visual system of human and nonhuman primates. Annu. Rev. Neurosci..

[89] Verhoef BE, Michelet P, Vogels R, Janssen P (2015). Choice-related activity in the anterior intraparietal area during 3-D structure categorization. J. Cogn. Neurosci..

[90] Murata A, Gallese V, Luppino G, Kaseda M, Sakata H (2000). Selectivity for the shape, size, and orientation of objects for grasping in neurons of monkey parietal area AIP. J. Neurophysiol..

[91] Sakata H, Taira M, Murata A, Mine S (1995). Neural mechanisms of visual guidance of hand action in the parietal cortex of the monkey. Cereb. Cortex (New York, NY).

[92] Taira M, Mine S, Georgopoulos AP, Murata A, Sakata H (1990). Parietal cortex neurons of the monkey related to the visual guidance of hand movement. Exp. Brain Res..

[93] Chouinard PA, Large ME, Chang EC, Goodale MA (2009). Dissociable neural mechanisms for determining the perceived heaviness of objects and the predicted weight of objects during lifting: an fMRI investigation of the size-weight illusion. Neuroimage.

[94] Goodale MA, Milner AD (1992). Separate visual pathways for perception and action. Trends Neurosci..

[95] Faul F, Erdfelder E, Lang AG, Buchner A (2007). G* Power 3: A flexible statistical power analysis program for the social, behavioral, and biomedical sciences. Behav. Res. Methods.

[96] Creem-Regehr SH, Lee JN (2005). Neural representations of graspable objects: are tools special?. Brain Res. Cogn. Brain Res..

[97] Hirschtick, J. K. *et al* OnShape.com: Multi-user cloud parametric feature-based 3D CAD system. U.S. Patent 10,437,938 (2019).

[98] Fabbri S, Stubbs KM, Cusack R, Culham JC (2016). Disentangling representations of object and grasp properties in the human brain. J. Neurosci..

